# Genomics reveals zoonotic and sustained human mpox spread in West Africa

**DOI:** 10.1038/s41586-025-09128-2

**Published:** 2025-05-19

**Authors:** Edyth Parker, Ifeanyi F. Omah, Delia Doreen Djuicy, Andrew Magee, Christopher H. Tomkins-Tinch, James Richard Otieno, Patrick Varilly, Akeemat Opeyemi Ayinla, Ayotunde E. Sijuwola, Muhammad I. Ahmed, Oludayo O. Ope-Ewe, Olusola Akinola Ogunsanya, Alhaji Olono, Femi Mudasiru Saibu, Philomena Eromon, Moïse Henri Moumbeket Yifomnjou, Loique Landry Messanga Essengue, Martial Gides Wansi Yonga, Gael Dieudonné Essima, Ibrahim Pascal Touoyem, Landry Jules Mouliem Mounchili, Sara Irene Eyangoh, Alain Georges Mballa Etoundi, Linda Esso, Inès Mandah Emah Nguidjol, Steve Franck Metomb, Cornelius Chebo, Samuel Mbah Agwe, Hans Makembe Mossi, Chanceline Ndongo Bilounga, Olusola Akanbi, Abiodun Egwuenu, Odianosen Ehiakhamen, Chimaobi Chukwu, Kabiru Suleiman, Afolabi Akinpelu, Adama Ahmad, Khadijah Isa Imam, Richard Ojedele, Victor Oripenaye, Kenneth Ikeata, Sophiyah Adelakun, Babatunde Olajumoke, Áine O’Toole, Mark Zeller, Karthik Gangavarapu, Daniel J. Park, Gerald Mboowa, Sofonias Kifle Tessema, Yenew Kebede Tebeje, Onikepe Folarin, Anise Happi, Philippe Lemey, Marc A. Suchard, Kristian G. Andersen, Pardis Sabeti, Andrew Rambaut, Chikwe Ihekweazu, Idris Jide, Ifedayo Adetifa, Richard Njouom, Christian T. Happi

**Affiliations:** 1 African Center of Excellence for Genomics of Infectious Diseases, Redeemer’s University, Ede, Nigeria; 2 Department of Immunology and Microbiology, The Scripps Research Institute, La Jolla, CA USA; 3 Institute of Ecology and Evolution, University of Edinburgh, Edinburgh, UK; 4 Department of Parasitology and Entomology, Nnamdi Azikiwe University, Awka, Nigeria; 5 Virology Service, Centre Pasteur du Cameroun, Yaounde, Cameroon; 6 Department of Human Genetics, David Geffen School of Medicine, University of California Los Angeles, Los Angeles, CA USA; 7 The Broad Institute of MIT and Harvard, Cambridge, MA USA; 8 Theiagen Genomics, Highlands Ranch, CO USA; 9 Department for the Control of Disease, Epidemics and Pandemics, Ministry of Public Health, Yaounde, Cameroon; 10 Nigeria Centre for Disease Control and Prevention, Abuja, Nigeria; 11 Africa Centres for Disease Control and Prevention, Addis Ababa, Ethiopia; 12 Department of Biological Sciences, Faculty of Natural Sciences, Redeemer’s University, Ede, Nigeria; 13 Department of Microbiology, Immunology and Transplantation, Rega Institute, KU Leuven, Leuven, Belgium; 14 Department of Biomathematics, David Geffen School of Medicine, University of California Los Angeles, Los Angeles, CA USA; 15 Department of Biostatistics, Fielding School of Public Health, University of California Los Angeles, Los Angeles, CA USA; 16 Scripps Research Translational Institute, La Jolla, CA USA; 17 Department of Immunology and Infectious Diseases, Harvard T H Chan School of Public Health, Boston, MA USA

**Keywords:** Viral infection, Molecular evolution

## Abstract

Five years before the 2022 multi-country mpox outbreak, Nigeria and Cameroon reported their first cases in more than three decades^1,2^. Whereas the outbreak in Nigeria is recognized as an ongoing human epidemic, the drivers of the resurgence in Cameroon remain unclear^3,4^. The rate of zoonoses remains uncertain in both countries, and gaps in genomic data obscure the timing and zoonotic and geographic origin of monkeypox virus (MPXV) emergence in humans. Here, to address these uncertainties, we sequenced 118 MPXV genomes isolated from cases in Nigeria and Cameroon between 2018 and 2023. We show that in contrast to cases in Nigeria, cases in Cameroon are the result of repeated zoonoses, with two distinct zoonotic lineages circulating across the Nigeria–Cameroon border. Our findings suggest that shared animal populations in the cross-border forest ecosystems drive the emergence and spread of the virus. Accordingly, we identify the closest zoonotic outgroup to the Nigerian human epidemic lineage (hMPXV-1) in a southern Nigerian border state. We estimate that the shared ancestor of the zoonotic outgroup and hMPXV-1 circulated in animals in southern Nigeria in late 2013. We find that hMPXV-1 emerged in humans in August 2014 in the southern Rivers State and circulated undetected for three years. Rivers State was the main source of viral spread during the human epidemic. Our study sheds light on the recent establishment of MPXV in the human population and highlights the risk of persistent zoonotic emergence of MPXV in the complex border regions of Cameroon and Nigeria.

## Abstract (web summary)

Monkeypox virus genomic data from Nigeria and Cameroon, sampled between 2018 and 2023, indicate that the virus spread through repeated zoonoses in Cameroon, whereas in Nigeria, it spread mainly through human–human transmission, predominantly originating in Rivers State.

## Main

The viral zoonosis mpox is caused by infection with the *Orthopoxvirus* MPXV, transmitted from an unknown animal reservoir^5,6^. MPXV diversity is partitioned into two major clades. Clade I was historically restricted to animal populations in Central Africa, and clade II with its subclades IIa and IIb was restricted to West Africa^5,6^. From first identification in humans in 1970 to 2017, mpox cases were largely infrequent, found in rural endemic regions, and exhibited limited human-to-human transmission^1,7,8^. However, a marked increase in cases has been observed in both endemic and non-endemic countries in recent years^9^. Notably, Nigeria and Cameroon reported their first cases in more than three decades in 2017 and 2018, respectively^1,2,4,10^.

In May 2022, a clade IIb lineage denoted B.1 rapidly disseminated around the world to cause the multi-country mpox outbreak^11^. The apparent human-to-human transmission of the B.1 lineage raised the possibility of a new MPXV transmission route. B.1 showed significant divergence from the closest clade IIb genome sampled in Nigeria in 2018, with an evolutionary rate higher than the expected rate for Orthopoxviruses^12^. The multi-country outbreak was characterized by enrichment of mutations in a dinucleotide context associated with the cytosine deaminase activity of the APOBEC3 host antiviral mechanism^3,13^. This mutational signature has not been observed in sequences from zoonotic infections, suggesting that APOBEC3 genomic editing was a feature of sustained transmission in the human population^3^.

In light of this new evolutionary dynamic, several studies confirmed that the ongoing mpox epidemic in Nigeria was driven by sustained human-to-human transmission^3,14^. It is estimated that MPXV emerged in the human population in Nigeria in 2016, circulating and diversifying cryptically into multiple distinct lineages^3,14^. By contrast, the recent increased incidence of mpox in Cameroon was most probably driven by sporadic cases and limited outbreaks resulting from zoonotic transmission^4^. The contiguous Cameroonian Highlands forest belt, spanning Nigeria and Cameroon, provides suitable habitats for animal reservoirs^15,16^. Agriculture, hunting and human settlements in forested areas of this region increase interaction at the human–animal interface and the risk of zoonotic transmission^2,4^. Human movement across the porous border can also facilitate viral spread, especially as outbreaks in both countries intensify^2^.

Limited availability of full-length MPXV genomes from the region, however, means that there are currently several unanswered questions about the zoonotic and human transmission dynamics of MPXV^14^. The extent of human-to-human transmission in Nigeria and Cameroon, as well as the role of zoonotic spillover events in both countries remain unclear. The drivers and patterns of the sustained human epidemic in Nigeria are also unknown. Enhancing our understanding of the cross-border MPXV zoonotic transmission dynamics is essential to assess the risk of recurrent spillover events and bidirectional transmission. Concurrently, a deeper insight into MPXV transmission in human epidemics is needed to inform public health interventions that reduce local cases and limit viral export. To support these goals, we established a pan-African consortium to compile the largest MPXV dataset thus far.

## Zoonoses drove resurgence in Cameroon

**Fig. 1:**
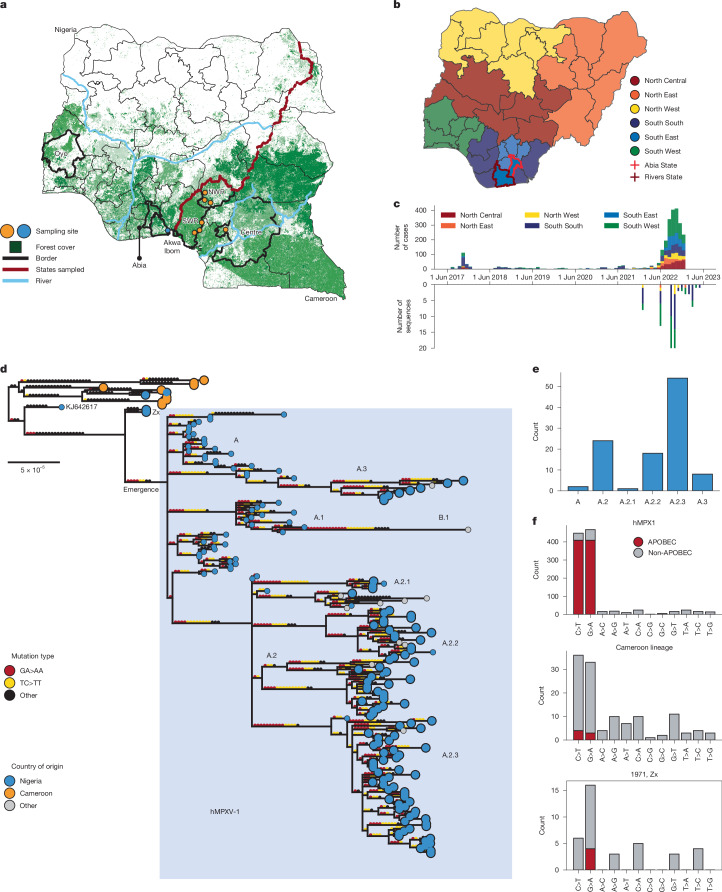
Drivers of mpox cases in Cameroon and Nigeria. **a**, Map of Nigeria and Cameroon, showcasing the ecological setting of zoonotic MPXV. The forest cover is highlighted in green. The border between Nigeria and Cameroon is annotated in red, with the Niger River in Nigeria and Sanaga River in Cameroon highlighted in light blue. Our sampling sites in Cameroon are annotated in orange, and states of interest are annotated with highlighted borders. Sampling sites of zoonotic Nigerian sequences are annotated in blue. NWR, Northwest Region of Cameroon; SWR, Southwest Region of Cameroon. **b**, Geopolitical regions of Nigeria, with Abia State and Rivers State highlighted with red borders. **c**, Epidemiological incidence of mpox cases in Nigeria coloured by geopolitical region (top) relative to the temporal and geographic distribution of the Nigerian genomic dataset (bottom). **d**, Clade IIb phylogeny with reconstructed SNPs mapped onto branches. We performed ancestral state reconstruction across our clade IIb phylogeny to map SNPs to their relevant branches. We annotated APOBEC3 characteristic mutations (CT or GA) in the correct dimer context along branches and calculated their relative proportion across internal branches (**f**). APOBEC3 mutations along the branches are annotated in yellow and red, with the remainder in grey and black. The hMPXV-1 clade (lineage A) is highlighted in the light blue box, with lineage annotation in text. Our new zoonotic outgroup sequences are annotated as Zx. Our sequences (*n* = 118) are highlighted as enlarged tips. The lineage sampled from Cameroon and Akwa Ibom in Nigeria is annotated in orange. **e**, Lineage distribution of our hMPXV-1 sequences. **f**, The number of APOBEC3 SNPs among all mutations for the zoonotic lineage from Cameroon and Akwa Ibom, the remaining zoonotic subtree (KJ642617 and Zx, annotated in **d**) and the hMPXV-1 subtree (highlighted and annotated in **d**).

To resolve the relative contributions of sustained human-to-human and zoonotic transmission in the two countries, we generated 118 near-complete MPXV genomes sampled from Nigeria (*n* = 109) and Cameroon (*n* = 9) between 2018 and 2023. The majority of our Cameroonian sequences were sampled from the rural Southwest and Northwest regions bordering Nigeria, which account for the second and third highest number of mpox cases in Cameroon, respectively (Fig. 1a). The Nigerian sequences were sampled predominantly from the South South, South East and South West regions (Fig. 1b). Southern states were the epicentre of the epidemic from 2017 onwards, and reported the earliest cases (Fig. 1c). From 2022 onwards, both northern and southern Nigeria experienced a substantial resurgence of cases after a period of low incidence (Fig. 1c). Our Nigerian dataset largely encompasses the resurgence from 2022 onwards (Fig. 1c). All of the sequences in the dataset belonged to clade IIb.

To quantify the relative extent to which sustained human transmission and zoonotic spillover events contribute to ongoing mpox cases in both countries, we reconstructed the clade IIb phylogeny with all available clade IIb sequences. We found that 105 of the 109 Nigerian sequences were interspersed throughout 6 divergent, co-circulating sublineages of the human-to-human transmitting lineage in Nigeria termed hMPXV-1 (ref. ^14^) (Fig. 1d,e). The divergence of the sublineages suggests that hMPXV-1 has cryptically circulated and diversified, driven largely by APOBEC3 activity, in the human population in Nigeria for a prolonged period after initial spillover^3,14^ (Fig. 1d). Within hMPXV-1, distinct lineages are designated according to a system similar to the SARS-CoV-2 Pango nomenclature^17,18^. Under this nomenclature, hMPXV-1 is referred to as lineage A, with its descendants designated as, for example, A.1, and second subdivision descendants are designated as, for example, A.1.1 (refs. ^3,14,18^). The majority of the 105 sequences belonged to sublineage A.2.3 (Fig. 1e). None of the 105 sequences were from lineage A.1.1, from which the B.1 multi-country outbreak lineage descended (Fig. 1d). Only 4 of the 109 sampled cases in Nigeria were identified as probable zoonotic infections (around 3.7%), as their sequences did not fall within the hMPXV-1 lineage (Fig. 1d). Collectively, these findings indicate that sustained human transmission is the primary driver of mpox cases in Nigeria.

Conversely, we found that all nine Cameroonian sequences form a divergent basal sister lineage to hMPXV-1 and its zoonotic outgroup KJ642617 (Fig. 1d). This suggests that the sampled cases in Cameroon were the result of zoonotic transmission and are not linked to hMPXV-1. To confirm this, we quantified the APOBEC3 mutational bias characteristic of human-to-human transmission across our phylogeny (Methods). We observed a significant proportion of mutations consistent with APOBEC3 activity across the internal and terminal branches of hMPXV-1, including our new sequences (Fig. 1d,f). Approximately 74% of reconstructed single nucleotide polymorphisms (SNPs) in hMPXV-1 were indicative of APOBEC3 editing, consistent with previous work^3^. This APOBEC3 enrichment confirms that 105 of the 109 Nigerian sequences are a result of the sustained human transmission. By contrast, we found that only 9% of reconstructed SNPs across the remaining parts of the tree were APOBEC3-type mutations (Fig. 1d,f). This includes all nine Cameroonian sequences and the four probable zoonotic infections from Nigeria. Overall, we sampled 13 zoonotic infections (around 11%) in our total dataset. Our findings suggests that all (100%) of the sampled Cameroonian cases resulted from zoonotic transmission, confirming that that the recent surge in cases was not driven by sustained human-to-human transmission as observed in Nigeria^3,4^. Our findings also corroborate the continued if minor role of zoonotic transmission in the epidemiology of mpox in Nigeria (approximately 3.7% of sampled cases).

## Distinct zoonotic lineages cross the border

The Nigeria–Cameroon border is covered by a complex forest belt that extends into both countries and hosts many animal populations that are susceptible to MPXV infection and can move freely across borders^15,16^ (Fig. 1a). The forest belt also hosts a substantial amount of subsistence hunting and wild game trade across borders, alongside high levels of cross-border human movement that may drive viral spread between the two countries^2,19–21^. Leveraging our dataset encompassing both countries, we sought to investigate cross-border zoonotic transmission to elucidate the potential drivers of bidirectional spread.

In our phylogeny (Fig. 1d), we found that two of the four Nigerian zoonotic sequences clustered with Cameroonian sequences in the newly sampled sister lineage to hMPXV-1 (refs. ^3,14^) (Fig. 1d). This indicates that the new zoonotic lineage is likely to have disseminated across the border. The two Nigerian sequences were from the southern Akwa Ibom state adjacent to Cross River State, which shares a border with the regions from which our Cameroonian cases were sampled (Fig. 1a). The Akwa Ibom sequences were most closely related to a sequence we sampled in Mbongue in the Southwest Region of Cameroon, although they diverged substantially (separated by 25 and 29 SNPs from the Mbongue sequence; Fig. 1d). Together, the phylogeny suggests that this new lineage, with its long internal branches, represents independent zoonotic transmissions from a viral population that must have diverged over a long period of time in a shared animal population, driving bidirectional transmission from the cross-border forest ecosystem. Furthermore, before this study the only high-quality zoonotic clade IIb sequence was KJ642617, the closest zoonotic outgroup to hMPXV-1 (Fig. 1a). KJ642617 was sampled in 1971 in Abia province in southern Nigeria (Fig. 1a,b,d). KJ642617 and the new lineage, predominantly sampled in Cameroon, share a common ancestor that must also have circulated in the animal population of the cross-border ecosystem several decades ago (Fig. 1d).

**Fig. 2:**
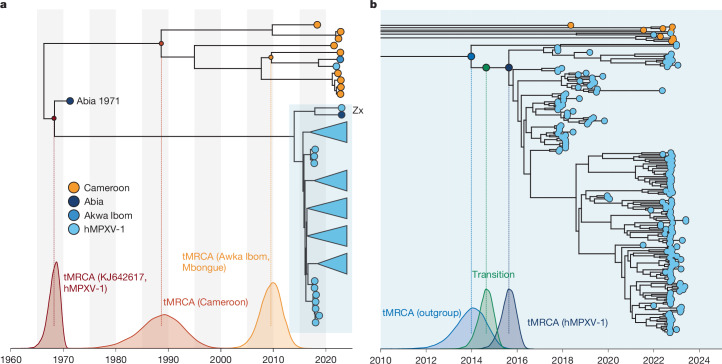
Time-resolved global phylogeny of clade IIb. **a**, The time-resolved global phylogeny of clade IIb. The new zoonotic outgroup from Abia is annotated as Zx. The distribution along the *x* axis represents the tMRCA of the node. Sublineages of lineage A in hMPXV-1 are collapsed for visualization. **b**, Detailed view of the Zx outgroup and hMPXV-1, showing timing of outgroup circulation, transition to sustain human transmission and tMRCA (hMPXV-1).

As the relationship of KJ642617 to the new zoonotic lineage and the nested Akwa Ibom sequences support historic and recent viral dissemination across the border, we performed Bayesian phylogenetic reconstructions to investigate the timing of the cross-border dissemination^3^. In our reconstructions, we found that the Cameroonian lineage and KJ642617 shared a common ancestor that circulated in the animal reservoir around February 1966 (median time to the most recent common ancestor (tMRCA); 95% highest posterior density (HPD): February 1963 to November 1968; Fig. 2a). There is also evidence of more recent cross-border viral spread, as the Akwa Ibom sequences diverged from the Mbongue sequence in July 2009 (95% HPD: June 2006 to June 2013; Fig. 2a). Sparse sampling limits our ability to definitively resolve the geographic origin of the common ancestor of all of clade IIb. However, the country of origin and direction of spread is less meaningful for a virus in a freely moving animal population in a cross-border ecosystem where wild game sources and markets are often shared across the borders. Together, our zoonotic sequences support both recent and historic viral spread across a porous Nigeria–Cameroon border, probably originating in a shared animal population hosting considerable diversity in the cross-border forest ecosystem^5,22–26^.

## Zoonotic ancestor traced to south Nigeria

Building on our previous findings of a dynamic reservoir in the cross-border forest ecosystem, we aimed to identify the potential zoonotic ancestor of hMPXV-1 by leveraging our dataset from the forested border regions. The genomic data support a single zoonotic origin for hMPXV-1 (ref. ^3^). However, no closely related zoonotic ancestor has been identified for hMPXV-1 thus far, with the closest known zoonotic outgroup (KJ642617) having been sampled in Nigeria in 1971 (Fig. 1a,b,d). Notably, we found that our two remaining Nigerian zoonotic sequences formed a sister lineage to hMPXV-1 (denoted Zx in Fig. 1d), breaking up the long stem branch from KJ642617 to hMPXV-1 (Fig. 1d). As our new Zx sequences share a direct common ancestor with hMPXV-1, they represent the closest zoonotic outgroup to hMPXV-1.

The Zx sequences reduced the stem branch from a zoonotic ancestor to hMPXV-1 from 27 to 8 SNPs. The more recent divergence between our new Zx outgroup and hMPXV-1 provides a narrow timeframe for when the zoonotic ancestor of hMPXV-1 was circulating in animals, as well as for when hMPXV-1 first emerged in humans. To estimate these timings with our newly identified outgroup, we adopted a partitioned two-epoch model described previously^3^, implemented in the BEAST software package, which models APOBEC3-mediated evolution by allowing for a transition from a polymerase-error-driven evolutionary rate to an APOBEC3-driven rate across the tree in a partitioned alignment (Methods). In our Bayesian reconstructions, we estimated that the Zx outgroup shared a common ancestor with hMPXV-1 that circulated in an animal population in late November 2013 (95% HPD: July 2012 to March 2015) (Fig. 2b).

With this additional phylogenetic information for the Zx outgroup, we also estimated that the transition to sustained human transmission, representing the time of emergence in the human population, occurred in August 2014 (95% HPD: 3 December 2013 to 25 March 2015) (Fig. 2b). Our estimate is about 13.5 months earlier than previous reports, although the credible intervals overlap^3^. When the highly informative Zx sequences are excluded from the phylogeny, our estimate for the transition to sustained human-to-human transmission shifts to mid-September 2015 (95% HPD: January 2015 to May 2016), aligning closely with the previous estimates^3^. We estimated that the tMRCA of hMPXV-1 was August 2015 (95% HPD: 12 December 2014 to 18 March 2016), representing the time at which hMPXV-1 started to diversify in humans (Fig. 2b). Our estimate is approximately seven months earlier than the previous estimate, although the credible intervals overlap^3^.

Notably, our Zx sequences were sampled in southern Abia State close to Cross River and Akwa Ibom State bordering Cameroon, where the previous zoonotic outgroup KJ642617 was also sampled in 1971. As both zoonotic outgroups were sampled in Abia over a 50-year time span, this suggests that the precursor lineage of hMPXV-1 may have circulated in an animal population in the south for decades before the recent emergence. In our Bayesian reconstructions, we found that the Zx zoonotic outgroup diverged from KJ642617 in early 1968 (95% HPD: January 1966 to March 1970) (Fig. 2a). This suggests that the hMPXV-1 precursor lineage circulated in an animal population in Abia or southern Nigeria for around 50 years.

Together, our findings indicate that the ancestor of hMPXV-1 circulated in animals in the southern state Abia for less than a year before emergence. This is consistent with the epidemiological data, which show that the southern states were the epicentre of the early stages of the Nigerian mpox outbreak (Fig. 1c). On emergence, hMPXV-1 circulated cryptically in the human population in Nigeria for approximately three years before detection in September 2017, and for more than seven years before disseminating globally during the B.1 multi-country outbreak in 2022.

## Viral spread from southern Nigeria

**Fig. 3:**
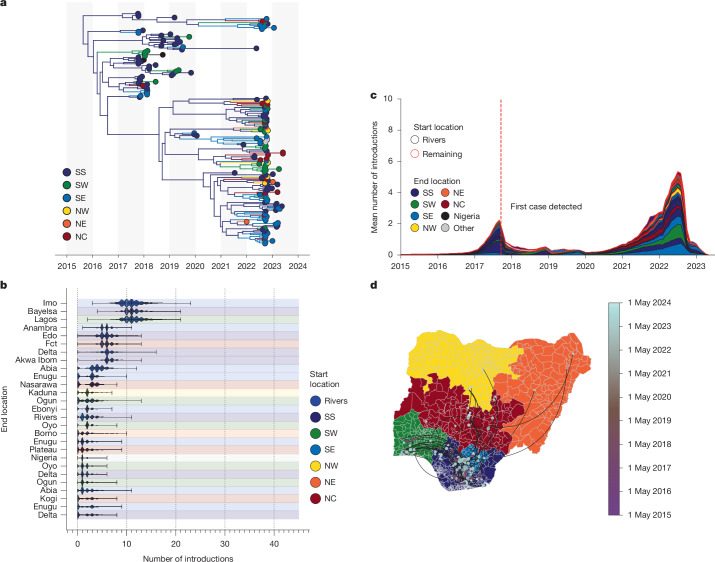
Spatiotemporal spread of hMPXV-1 in Nigeria. **a**, Phylogeographic reconstruction of the spatiotemporal spread of clade IIb in Nigeria. The branches of the maximum clade credibility tree are coloured by source region. **b**, Distribution of total number of introductions by state from each start location summarized across the posterior of 10,000 trees to each end location on the *y* axis. The distribution represents the 95% HPD. The regions of the end location are indicated by background colour. **c**, Distribution of the number of introductions over time by state, summarized across the posterior of 10,000 trees. The end location is coloured by region. The start location is highlighted by transparency. **d**, Continuous phylogeography of hMPXV-1 spatiotemporal spread across Nigeria, with timing of viral dissemination indicated by colour. NC, North Central; NE, North East; NW, North West; SE, South East; SS, South South; SW, South West.

**Extended Data Fig. 1:**
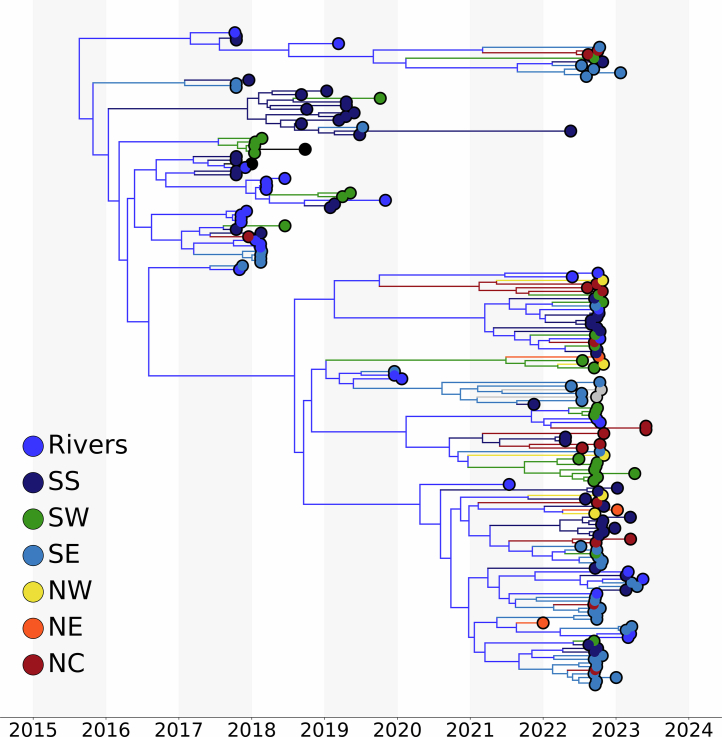
Phylogeographic analyses of Clade IIb in Nigeria. The branches of the MCC are coloured by source state, as per legend. Non-Rivers state were grouped by region. SS: South South; SW: South West; SE: South East; NW: North West; NE: North East’ NC: North Central.

Our results support that the zoonotic progenitor of hMPXV-1 probably circulated in southern Nigeria, in the forested border regions. Nevertheless, the precise geographic origin of the emergence and the subsequent outbreak of hMPXV-1 remains undetermined. To explore this, we used discrete and continuous phylogeographic reconstructions on a state and regional level. We found that both of the reconstructions support that hMPXV-1 probably originated in Rivers State in the South South region (posterior = 0.97; Fig. 3a and Extended Data Fig. 1). Notably, this is consistent with our sampling of the closest zoonotic outgroup in the neighbouring southern Abia State and the epidemiological data (Fig. 1a,c).

**Extended Data Fig. 2:**
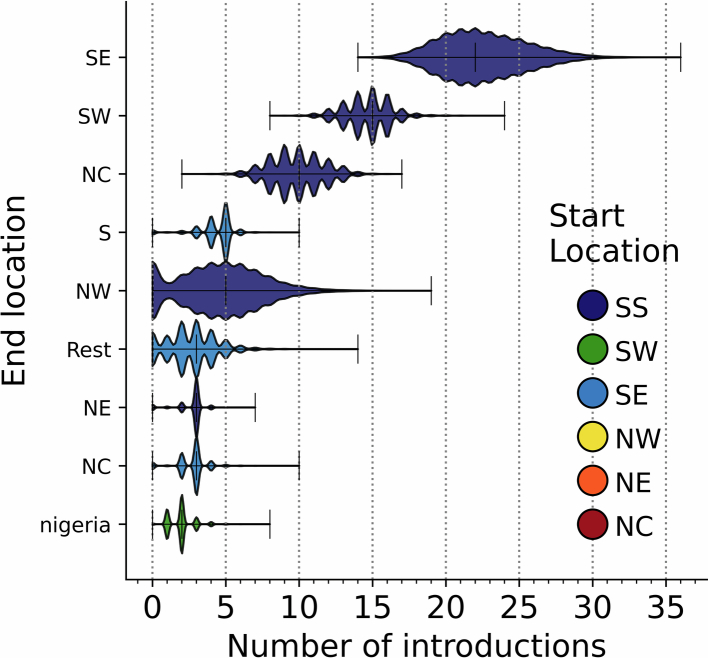
Total number of introductions by region from each start region. Data are summarised across the posterior of 10 000 trees, annotated as per legend in colour, to each end location on the y-axis.

The epidemiological data indicate that the southern states of Nigeria were the early epicentre of the mpox epidemic, with the northern states only reporting a large number of cases after the resurgence in 2022 (Fig. 1a). However, it is not known whether there was under-ascertained and unsampled transmission outside of the southern states before the resurgence. It is also unclear which states contributed to interstate viral dissemination and how these patterns may have shifted across the different phases of the epidemic. We used our phylogeographic reconstructions to investigate these spatiotemporal dynamics of hMPXV-1 in Nigeria. We found that Rivers State was also the primary source of interstate viral exports across the epidemic, with an estimated 75 viral introductions originating in Rivers (95% HPD: 70–84) (Fig. 3b and Extended Data Fig. 1). The highest number of viral exports from Rivers spread to other South South states, followed by the South East and South West (Fig. 3b and Extended Data Fig. 2). Overall, neighbouring Imo, Bayelsa and Lagos states in the South West region had the highest number of introductions from Rivers. The remainder of the South South states, as well as the South East and South West states, were all equally the second highest source of viral exports overall.

**Extended Data Fig. 3:**
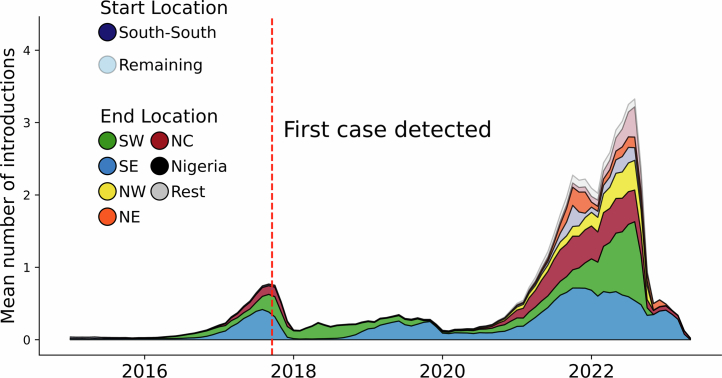
The distribution of the number of introductions across time by region. Data are summarised across the posterior of 10 000 trees. Distribution represents the 95% highest posterior density. The end location state is coloured by region, as per legend. The start location is highlighted by transparency: all introductions originating from the South-South region are presented with no transparency, whereas introductions originating from other regions are transparent.

We found that all introductions in the early epidemic originated in Rivers State (Fig. 3c). Viral spread from Rivers into neighbouring South South states, such as Bayelsa and Imo, as well as Lagos in the South West region, occurred as early as 2016 (Fig. 3c and Extended Data Fig. 3). This is consistent with the epidemiological data, with the first case reported in southern Bayelsa on 11 September 2017 (Fig. 1c). All but one of the sampled introductions into northern states occurred in the later phase of the epidemic, after the resurgence towards 2022 (Figs. 1c and 3c). This spatiotemporal pattern was consistent across our discrete and continuous phylogeographic reconstructions on both a regional and a state level (Fig. 3d and Extended Data Figs. 1–3).

To investigate how widespread hMPXV-1 transmission was by the time the outbreak was declared on 22 September 2017, we performed a continuous phylogeographic reconstruction. We found that the virus had spread more than 500 km beyond Rivers State into Bayelsa, Imo, Delta, Edo, Federal Capital Territory and Lagos states before the first case was detected on 11 September 2017 (Fig. 3d). Collectively, this suggests that the human epidemic originated in Rivers State, with early spread of the virus to neighbouring South South and South East states and Lagos before the outbreak declaration, and with delayed dispersal to the north.

**Fig. 4:**
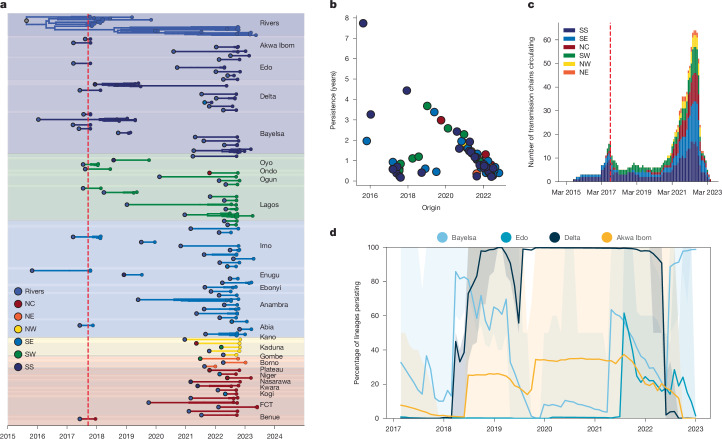
Transmission dynamics of hMPXV-1 in Nigeria. **a**, Persistence of transmission chains across all sampled Nigerian states. Individual chains are coloured by region, with the boundary of each individual state highlighted by a filled background and annotated in text on the right. The start of each transmission chain is coloured by its state of origin. The dashed red line indicates the date on which the first case in was reported in Bayelsa (17 September 2017). **b**, The persistence of each transmission chain versus time of origin, summarized across the posterior of 10,000 trees, coloured by region. Colour scheme as in **c**. **c**, The number of transmission chains circulating across all regions over time, calculated using a one-month sliding window. The dashed red line indicates the date on which the outbreak was declared (22 September 2017). **d**, The percentage of transmission chains that persist over time for South South states, excluding Rivers. Colour bands represent 95% HPD.

**Extended Data Fig. 4:**
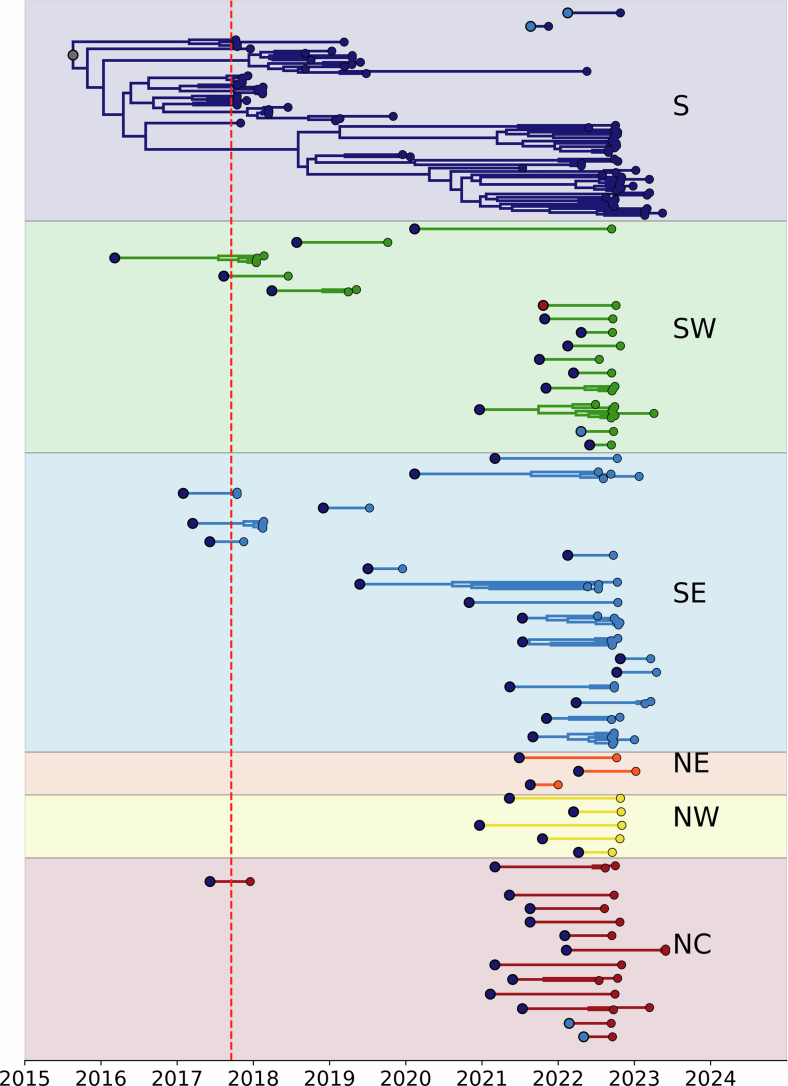
Persistence of transmission chains across all regions. As annotated in text. The start of each transmission chain is coloured by its region of origin. The red line indicates the date of report for the first case in Bayelsa.

Although our findings suggest that River State was the dominant source of viral exports, it is not clear whether cases across Nigerian states were continuously seeded by re-introduction from Rivers, or whether there were also locally persistent transmission chains in other regions sustaining local epidemics. To understand the respective contribution of persistence and introductions, we investigated the persistence of transmission chains in each state. We found that hMPXV-1 has persistently circulated in Rivers from emergence onwards (Fig. 4a,b). hMPXV-1 diversified in Rivers State for more than two years before the first case was reported in Bayelsa^1,10^ on 11 September 2017 (Fig. 4a and Extended Data Fig. 4). When the outbreak was declared, transmission chains had already been established in 11 states outside Rivers (Fig. 4a). Delta and Bayelsa in the South South region had the second longest persistence of a transmission chain at approximately 4.5 and 3 years, followed by the earliest chain established in Lagos (Fig. 4a,b). Outside of Rivers State and the early chains in Delta and Lagos, the longest persistence was estimated for lineages that were introduced during the period of low reported incidence in 2018–2021, when sampling was sparse (Figs. 1c and 4b).

We found that persistence was the primary driver of the epidemic in Rivers State, relative to repeat introductions (Fig. 4a,c). However, the percentage of transmission chains that were persistently circulating was dynamic over time in other South South states (Fig. 4d). Transmission chains seeded by Rivers State early in the epidemic in the South South, South West and South East regions persisted locally for less than two years, excluding the first transmission chains established in Bayelsa, Delta and Enugu (Fig. 4a,b). There was a significant increase in the number of transmission chains circulating during the resurgence of cases towards 2022 (Fig. 4c). This is consistent with the increased viral exports during this period (Fig. 3c), seeding new transmission chains that drove local surges across Nigeria (Figs. 1c and 4a). Transmission chains from the later stage of the epidemic were predominantly introduced from Rivers, and persisted for less than two years (Fig. 4a). However, it is unclear whether this pattern persists past the end of our sampling frame. There was no evidence for significant persistence in northern states before the later phase of the epidemic, which is consistent with the low reported incidence and delayed viral spread observed (Figs. 1c, 3d and 4a). Together, this further supports that Rivers State was the persistent source for the epidemic and local epidemics in other states were driven mainly by repeat introductions.

To account for uneven sampling across states, we also performed our phylogeographic analyses at the regional level. We observed a consistent pattern in our state-level analyses: early and predominant spread from the South South, with initial spread to the South East and South West. There was strong evidence of persistent circulation in the South South, with local epidemics in all other regions being driven by repeated introduction from the South South (Extended Data Figs. 1–4).

## Rivers State was the main source of spread

Our phylogeographic reconstructions consistently support a spatiotemporal pattern of early viral spread between and then from southern states. To identify potential drivers of this pattern, we used a phylogeographic generalized linear model that integrates covariates of spatial spread to determine what factors were associated with hMPXV-1 dispersal. We incorporated covariates in our model, including epidemiological, demographic, geographic and economic variables, as well as location-specific predictors that capture a pairwise binary transition across different states (Extended Data Table 1).

**Fig. 5:**
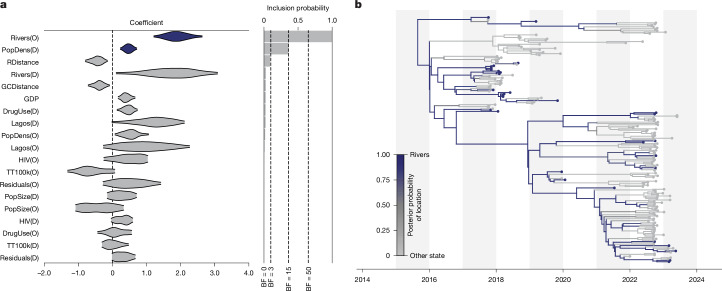
Drivers of spatiotemporal patterns of hMPXV-1 in Nigeria. **a**, Generalized linear model coefficients for spatial spread covariates, with corresponding Bayes factors. Significant covariates are highlighted in purple. Covariates are defined in Extended Data Table 1. **b**, Phylogeographic reconstruction of the migratory pattern of hMPXV-1, showing Rivers State as the origin (highlighted in purple) and other Nigerian states are shown in grey, in accordance with the posterior probability of location.

From the 19 covariates that we analysed, we found that the main predictor that positively affected viral dispersal was whether the lineage originated in Rivers State (Bayes factor > 50) (Fig. 5a,b). There was no support for the residual covariates that assessed the deviations of sampling numbers relative to epidemiological cases (Fig. 5a). This suggests that the ‘out-of-Rivers’ pattern is robust and was not a result of sampling heterogeneity across locations. This finding supports our previous analyses highlighting the early and dominant role of Rivers State in the spread of hMPXV-1 from emergence onwards (Fig. 5b). The population density in the destination was also positively associated with hMPXV-1 dispersal (Bayes factor > 15), which is consistent with the principles of a gravity model in epidemiology^27,28^ (Fig. 5a).

## Discussion

The ongoing zoonotic transmission in the forested border regions of Nigeria and Cameroon identified in this study underscores the continuous risk of MPXV emergence and/or re-emergence. Furthermore, this risk is likely to remain markedly underestimated owing to under-ascertainment of the number of cases and sparse genomic data. Concurrently, consensus evidence now strongly supports that MPXV clade IIb is no longer a solely zoonotic disease, but is sustained in a human subpopulation in Nigeria, where it has been circulating cryptically for nearly a decade. The drivers behind this emergence in humans remain uncertain, however. It is unclear why the initial zoonotic transmission event did not simply result in a self-limiting case or transmission chain as for all previous zoonotic infections. It is most likely that the zoonotic transmission occurred in a more interconnected, mobile subpopulation with greater connectivity to densely populated urban areas and more probable onward transmission driven by behavioural or demographic factors, as observed in the multi-country B.1 outbreak and the recent outbreak in the eastern Democratic Republic of the Congo^11,29^.

In this study, all of our Cameroonian sequences were sampled from individuals living in rural areas in the Southwest and Northwest regions, whereas our four zoonotic sequences from Nigeria were sampled in the southern states of Akwa Ibom and Abia. The Northwest and Southwest regions of Cameroon are dominated by the Cameroonian Highlands and Lower Guinean forest ecosystems, which extend into southern Nigeria^15^. These cross-border forest ecosystems are areas of high biodiversity and host a wide range of hosts that are potentially susceptible to MPXV^5,22–26^. In these regions, agricultural activities, subsistence hunting, the consumption and trade of wild game, and human settlements and movement in forested areas owing to internal displacement associated with conflict heighten exposure at the human–animal interface^2,4^. Our findings indicate that the cross-border forest ecosystems are likely to host the shared reservoir that drives viral dispersal between the countries. It is also likely that the extant clade IIb originated in this region. In light of this, improved surveillance in the wildlife population in the forest systems is needed to better understand the transmission and maintenance of MPXV in animal hosts. Additional sampling is likely to reveal substantial unsampled viral diversity in the reservoir, as observed in the deep divergences just in the sampled tree. It is also likely that enhanced surveillance in the human population in border-adjacent communities at the animal–human interface will reveal more zoonotic transmission events. Our results should therefore be interpreted within the limits of our sample, which represents only 4.2% and 7.5% of all suspected mpox cases in Nigeria and Cameroon, respectively.

Our study provides critical insights to facilitate strategic public health interventions in the human epidemic in West Africa. Across our phylogeographic reconstructions, we found that Rivers State and other South South states in Nigeria served as early, dominant and persistent sources of viral export. Interventions should accordingly be targeted to these regions. Notably, we found evidence of prolonged cryptic circulation and geographic expansion before detection in these regions, emphasizing the need for enhanced surveillance and improved diagnostic and surveillance infrastructure. Enhanced surveillance of cases is needed to characterize the underlying transmission network and associated risk factors, allowing targeted interventions before the epidemic becomes more generalized.

However, controlling ongoing mpox epidemics in Africa is impeded by inequities of access to resources such as diagnostics, vaccines and therapeutics^30,31^. Without access to therapeutics and vaccines, transmission cannot be reduced in either the sustained human epidemic or in populations at high risk for recurrent spillovers from the reservoir. Ongoing zoonotic and human transmission in Africa not only increases the probability of re-emergence and future multi-country outbreaks but, crucially, also results in preventable morbidity and mortality in endemic countries. The global community can no longer afford to neglect mpox in Africa or perpetuate inequities in therapeutic access in our vulnerably connected world.

## Methods

### Ethics declaration

No ethical approval was required for this study, as it is based on data from Nigeria’s national surveillance programme, collected by the Nigeria Centre for Disease Control and Prevention (NCDC) and Cameroon’s national surveillance programme, for which Centre Pasteur du Cameroun is the National Reference Laboratory. Under both programmes, individual written or oral informed consent was obtained from all participants with suspected mpox. Informed consent for children was obtained from their parents or recognized guardians.

### Sampling

Samples from Nigeria were collected by laboratory personnel and local government area disease surveillance and notification officers equipped with appropriate personal protective equipment, adhering to the guidelines outlined in the NCDC National Monkeypox Public Health Response Guidelines^32^. Samples comprised: swabs from the exudate of vesicular or pustular lesions, lesion crusts obtained during the acute rash phase, whole blood collected in EDTA or plain, non-anticoagulated tubes. All samples were labelled with case information and stored at 2–8 °C during transport to either the NCDC National Reference Laboratory (Gaduwa Abuja) or the Central Public Health Laboratory (Yaba, Lagos). On arrival, the crusts and swabs were eluted, and the serum or plasma was separated from the red blood cells. Subsequently, these components were stored at ultralow temperatures of ≤−70 °C at the NCDC biorepository. Our samples are predominantly collected from the South South and South East regions, and do not include the period closer to the estimated emergence or the start of the epidemic in 2017. However, as southern regions had the highest number of cases throughout the epidemic from 2017 to the 2022 resurgence, it is unlikely that the geographic distribution of samples represents a strong sampling bias.

From 2018 to 2022, a total of 28 human mpox cases were identified by the national surveillance programme in Cameroon. Suspected cases were identified by community health workers or clinicians and samples collected by a rapid response and investigation team equipped with appropriate personal protective equipment under the guidance of regional centres for epidemic prevention and control and following the national guidelines for surveillance and response to mpox outlined by the the Department for the Control of Disease, Epidemics and Pandemics of the Cameroonian ministry of health. Cases were confirmed by standard and genotyping real-time PCR at the Centre Pasteur du Cameroun, which hosts the National Reference Laboratory for mpox in Cameroon^4^. From the total cases, we selected 10 for sequencing on the basis of clade genotyping by real-time PCR, cycle threshold (value < 30) and sample availability. Samples were screened for DNA concentration and quality (total DNA > 500 ng and absorbance ratio >1.8 at 260 nm/230 nm and 260 nm/280 nm). Samples were from maculopapular vesicles, skin crusts or blood.

### Genome sequencing

Enrichment bead-linked transposomes were used to tagment the extracted DNA and enriched using Illumina RNA Prep enrichment with the viral surveillance panel. Libraries were quantified using a dsDNA Broad Range Assay, normalized to a concentration of 0.6 nM and sequenced on the Illumina NovaSeq 6000 platform with a read length of 151-base-pair paired-end reads at the African Centre of Excellence for Genomics of Infectious Diseases based at Redeemer’s University, Ede, Nigeria.

### Genome assembly

We performed initial de novo assembly with the viral-ngs pipeline, followed by reference-based assembly with an in-house pipeline (https://github.com/broadinstitute/viral-pipelines)^33^. In brief, we mapped reads against a clade IIb reference genome (NC_063383, an early hMPXV-1 genome from Nigeria) with bwa-mem^34^, and called consensus using samtools^35^ and iVar^36^.

### Genomic dataset curation

We combined our 118 genomes with all high-quality, publicly available clade IIb MPXV genomes from GenBank (as of August 2023). We included a single representative of the multi-country outbreak lineage B.1, as it was not our primary focus. We also included a clade IIa outgroup (DQ011153) to root the tree. In total, our dataset consists of 199 sequences.

### Phylogenetic analysis

We aligned our dataset to the clade IIb reference genome (NC_063383) using the squirrel package (https://github.com/aineniamh/squirrel)^3^. The alignment was trimmed, and the 3′ terminal repeat region and regions of repetition or low complexity were masked. We also masked clustered mutations as identified with the quality control mode of the squirrel package.

We investigated the preliminary placement of our sequences in a phylogeny of all available mpox genome sequences from GenBank across clades. We reconstructed the complete MPXV phylogeny with IQ-TREE v2.0, under the Jukes–Cantor substitution model^37^. We identified three B.1 lineage sequences in our dataset. To investigate whether these sequences represented re-importations to Nigeria, we reconstructed a phylogeny with 769 B.1 genomes from GenBank. We confirmed that the sequences represented re-importations of B.1 into Nigeria and excluded them from subsequent analyses (data not shown).

We reconstructed a phylogeny for clade IIb alone under the same parameters as the global phylogeny. We rooted to DQ011153, a clade IIa outgroup and removed it from the tree. We collapsed all zero branch lengths. We performed ancestral state reconstruction with IQ-TREE2 across the clade IIb phylogeny^37^. We mapped all nucleotide mutations that occurred across the phylogeny to internal branches using tree traversal, excluding missing data. Furthermore, we catalogued the dimer genomic context of all C→T or G→A mutations, as described^3^. We classified our sequences into lineages using the nomenclature developed previously^18^ using Nextclade^38^.

**Extended Data Fig. 5:**
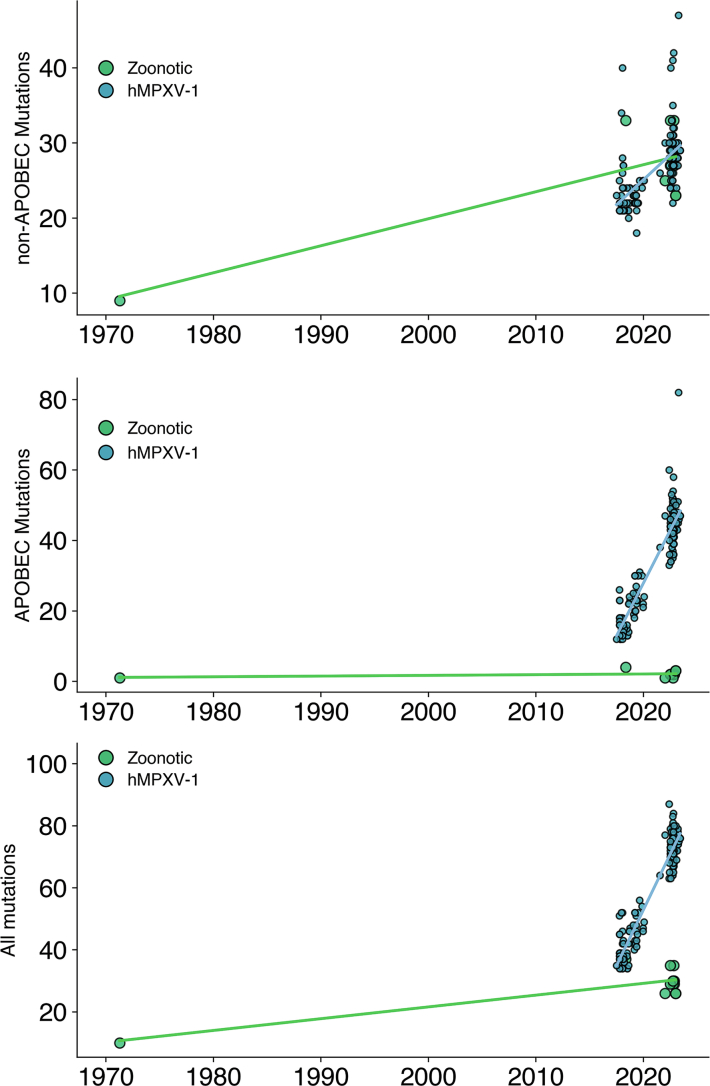
Root-to-tip regression of the accumulation of 1) non-APOBEC 2) APOBEC and 3) all mutations (APOBEC and non-APOBEC combined) across the human epidemic (hMPXV-1) and the zoonotic parts of the tree. Each data point represents a sequence in the tree. The hMPXV-1 subtree’s sequences are displayed in blue, and the zoonotic sequences in green. Lines represent the linear model fitted to the respective (hMPXV-1 and zoonotic) data.

**Extended Data Fig. 6:**
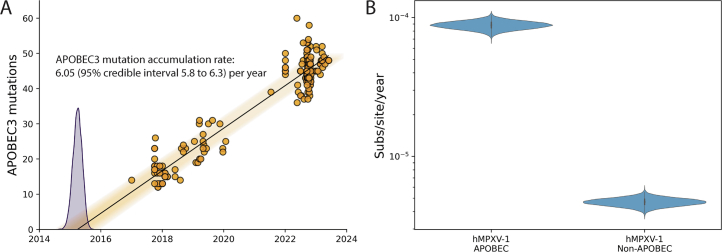
APOBEC and non-APOBEC driven clock rates. **A)** Root-to-tip regression of the accumulation of APOBEC3 mutations across the human epidemic. Line represents mean and error bands represent the 95% highest posterior densities **B)** Comparison of the evolutionary rates under the two-epoch partitioned model summarised across the posterior of 10 000 trees. Distribution represents the 95% highest posterior density, and boxplot represents 25th, 50th and 75% percentile.

Before Bayesian analyses, we performed temporal regression to evaluate the temporal signal at APOBEC and non-APOBEC sites (Extended Data Fig. 5). We also assessed the rate of APOBEC3 mutations accumulation per year across hMPXV-1 using a Bayesian regression on the root-to-tip data of the sequences as described^3^ (Extended Data Fig. 6a).

### Modelling APOBEC3-mediated evolution

We adopted a similar approach to one described previously^3^ to analyse the evolutionary dynamics of hMPXV-1 in the software package BEAST^39^ with the BEAGLE high-performance computing library^40^. First, we partitioned the clade IIb alignment into two distinct partitions, with a previously described custom script^3^. The first partition comprised sites with potential APOBEC3 modifications (specifically C→T and G→A substitutions in the dinucleotide context TC and GA), along with target sites (for example, C and G) that were conserved. In this partition, we masked all other sites as ambiguous nucleotides. This partition represents APOBEC3 mutations relative to the target APOBEC3 sites. The second partition inversely contained sites with the APOBEC3 target sites masked. The APOBEC3 alignment comprised 24,680 unmasked sites, whereas the non-APOBEC3 alignment comprised 172,529 sites. We used the standard nucleotide GTR+G substitution model with four distinct rate categories for the non-APOBEC3 partition. For the APOBEC3 partition, we developed a substitution process in which we categorize the nucleotides as modified (T) and unmodified (C). We used a two-state continuous-time Markov chain with an asymmetric rate to permit C→T mutations but not the reverse.

We used a two-epoch model to estimate the time of emergence of MPXV in the human population. Under this model, the evolutionary rate transitions from the background rate (that is, non-APOBEC3 rate, driven by polymerase error rate) to the APOBEC3 rate at a specific time point tp for the APOBEC3 partition. We parameterized this transition time as tp = tMRCA(lineage A) + *x*, where *x* is a free parameter in BEAST representing the pre-sampled transmission history before the most recent common ancestor of sampled lineage A viruses^3^. We incorporated a local clock to scale the mutation proportion attributed to APOBEC3 activity across the branches up to the transition time. We allowed the non-APOBEC3 partition to evolve under the background evolutionary rate across the entire phylogeny (see Extended Data Fig. 6b for rate estimates). We also used a two-phase coalescent model: the tree from the most recent common ancestor (lineage A) onward was modelled with an exponential growth model, with the earlier phase modelled as a constant-population size coalescent model. For each model, we ran 3 independent chains of 100 million states to ensure convergence, discarding the initial 10% of each chain as burn-in. The chains were then combined with LogCombiner. For all subsequent analyses, we assessed convergence using Tracer, and constructed a maximum clade credibility tree in TreeAnnotator 1.10 (ref. ^41^).

### Geographic history of hMPXV-1 in Nigeria

#### Discrete phylogeographic analysis

To investigate the spread of hMPXV-1 across Nigeria, we reconstructed the timing and pattern of geographic transitions across Nigerian states under an asymmetric discrete trait analyses^42^. We used Bayesian stochastic search variable selection to infer non-zero migration rates and identify statistically supported migration routes. We used a non-parametric skygrid coalescent tree prior, with 12 change points distributed over 10 years as described^43^. We combined three independent Markov chain Monte Carlo (MCMC) runs of 50 million states each, sampling every 2,000 states and discarding the respective initial 10% of trees as burn-in. We confirmed all effective sample size values were above 200.

We used a Markov jump counting procedure to investigate the timing and origin of geographic transitions, or Markov jumps, across the full posterior to account for uncertainty in phylogeographic reconstruction^44^. We used TreeMarkovJumpHistoryAnalyzer from the pre-release version of BEAST 1.10.5 to obtain the Markov jumps from posterior tree distributions^45^. Using the tree distribution annotated with Markov jumps, we performed the persistence analysis on a month-to-month interval to calculate the percentage of lineages that persisted in their ancestral state for each Nigerian state and region^45^. We used the PersistenceSummarizer from the pre-release version of BEAST 1.10.5.

We also performed all phylogeographic analyses on a regional level, to account for the uneven distribution of sequences across Nigerian states. We categorized the states into the six geopolitical zones of Nigeria^46^. We have a limited number of sequences from northern Nigeria, which had very low epidemic incidence between 2017 and 2022. To account for this, we combined the North West and North East zones into a single ‘North’ category. All trees were visualized using baltic (https://github.com/evogytis/baltic).

#### Continuous phylogeographic analysis

We performed a continuous phylogeographic analysis to quantify the dispersal of hMPXV-1 across Nigerian states. We assigned each sequence a latitude and longitude that matched the local government area or village of collection. We used the two-epoch and skygrid coalescent model described above, with a Cauchy distribution to model the among-branch heterogeneity in dispersal velocity^47^. We ran two independent MCMC chains of 50 million states, sampling every 2,000 states. We combined the chains after discarding 10% of the states as burn-in.

#### Discrete phylogeographic analysis using a sparse generalized linear model

To investigate the drivers of the transmission dynamics of the hMPXV-1 epidemic, we used a sparse generalized linear model in a continuous-time Markov chain diffusion framework^48^. We considered 19 covariates in the model, including epidemiological case counts relative to the numbers of sampled genomes per locations, demographic data, and geographic and economic factors (Extended Data Table 1). Other than the location-specific predictors, which were encoded as binary variables to reflect migration patterns, we log-transformed and standardized all other predictors. This standardization involved adding a pseudo-count to each entry to ensure a robust analysis^19^. We ran two independent MCMC chains of 50 million iterations, sampling every 2,000 iterations. We combined the resulting posterior distributions after removing the initial 10% as burn-in.

### Covariate collation

Covariates (Extended Data Table 1) included in the generalized linear model were collected from the following sources: epidemiological data was obtained from the Nigerian CDC; economic covariates were sourced from a previous study^49^; population covariates were sourced from the Bulletin of the National Bureau of Statistics^46^; HIV prevalence was obtained from the PEPFAR (US President’s Emergency Plan for AIDS Relief) programme^50^; and drug use statistics were obtained from the National Bureau of Statistics^51^. The distance by road travel between each states was calculated from using Maps.

### Geographic metadata

Administrative level 2 (admin2) metadata for the sampling location of sequences in the dataset were mapped to official admin2 as found in the Global Administrative Database (https://gadm.org). All shapes files were obtained from the United Nations Food and Agriculture Organization (FAO) geoNetwork (https://www.fao.org/land-water/databases-and-software/geonetwork/en/).

### Reporting summary

Further information on research design is available in the Nature Portfolio Reporting Summary linked to this article.

## Online content

Any methods, additional references, Nature Portfolio reporting summaries, source data, extended data, supplementary information, acknowledgements, peer review information; details of author contributions and competing interests; and statements of data and code availability are available at 10.1038/s41586-025-09128-2.

## Supplementary information

Reporting Summary

## Figures and Tables

**Extended Data Table 1:**
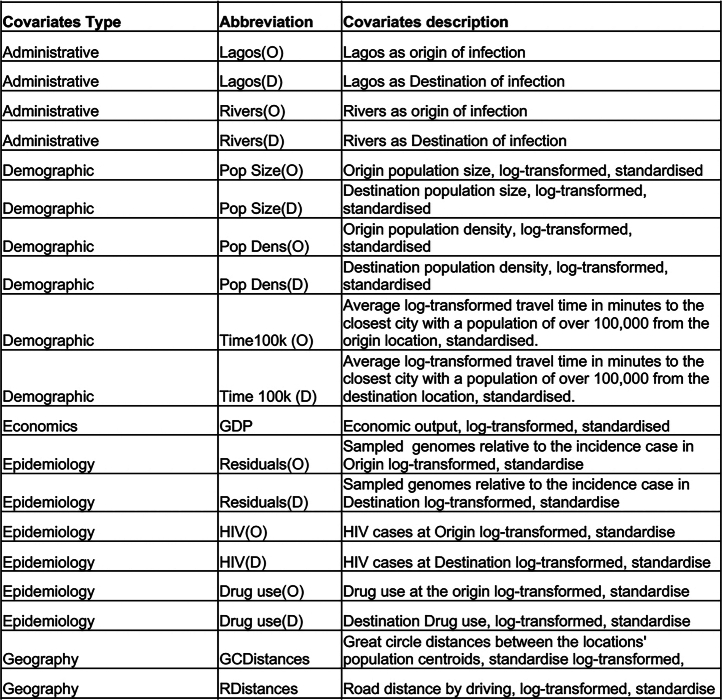
Covariates for GLM analyses

## Data Availability

All sequences are available on GenBank under accession numbers PP852943–PP853055. All other data are available at GutHub (https://github.com/andersen-lab/Mpox_West_Africa), excluding shape files, which are available at https://www.fao.org/land-water/databases-and-software/geonetwork/en/.

## Code availability

All code to run the analyses is available in https://github.com/andersen-lab/Mpox_West_Africa, excluding shape files which are available at https://www.fao.org/land-water/databases-and-software/geonetwork/en/.
